# Systemic Inflammatory Markers as Prognostic Factors in Oral Squamous Cell Carcinoma of the Tongue

**DOI:** 10.3390/biomedicines13030754

**Published:** 2025-03-20

**Authors:** Maria Giulia Cristofaro, Francesco Ferragina, Federico Tolino, Ida Barca

**Affiliations:** 1 Maxillofacial Surgery Unit, Department of Experimental and Clinical Medicine, Magna Graecia University of Catanzaro, 88100 Catanzaro, Italy; cristofaro@unicz.it (M.G.C.); barca.ida@gmail.com (I.B.); 2 Oral and Maxillofacial Surgery Unit, IRCCS University Hospital of Bologna, 40138 Bologna, Italy; federico.tolino@studenti.unicz.it

**Keywords:** oral tongue squamous cell carcinoma, hematological markers, neutrophil-to-lymphocyte ratio, platelet-to-lymphocyte ratio, oncological surgery

## Abstract

**Background**: Oral tongue squamous cell carcinoma (OTSCC) is a common disease that can cause occult metastasis (OM). **Methods**: This study aims to investigate the role of the pre-treatment neutrophil-to-lymphocyte ratio (NLR) in predicting the presence of neck OM in early-stage OTSCC. We reprocessed the pre-treatment blood data to calculate the NLR and the PLR on patients treated for OTSCC. We used a logistic regression model and the ROC curve to estimate the probability of metastases in cervical lymph nodes using data from pre-surgery blood tests. **Results**: During the period under review, 113 patients were treated for OTSCC; however, only 74 met the inclusion criteria and were, therefore, enrolled in the study. Twenty-five patients (35.3%) had lymph node metastases, and 46 (64.7%) did not. Without the NLR influence, the probability of metastasis is less than 50% (β0 = −1.058). A higher NLR value means a higher chance of metastasis. This is shown by the positive value of the NLR level coefficient (β1 = 0.135) and the ROC curve (AUC = 0.83). **Conclusions**: Our study showed a statistical correlation between high pre-treatment NLR values and neck OM in patients with OTSCC. These results may help to identify which patients are at risk of developing OM and then choose the right treatment.

## 1. Introduction

Oral tongue squamous cell carcinoma (OTSCC) is the most common malignancy in the oral cavity [1,2,3]. These tumors have the potential to invade locally and metastasize to the lymph nodes of the neck with an incidence described in the literature ranging from 8 to 46% [4,5]. Overall survival rates vary considerably between countries, with lymph node metastasis being the most significant factor influencing prognosis; lymph node metastasis identification is paramount for developing an effective therapeutic plan [5,6]. Despite the clinical negativity for lymph node involvement (cN0) observed in the early-stage OTSCC (T1–T2), the potential for generating occult metastasis (OM) remains. The prevalence of these lesions is estimated to be approximately 25% [7,8]. The elective neck dissection is an increasingly utilized surgical procedure for detecting lymph node OM in patients with cN0-OTSCC [9,10]. The five-year survival rate for patients with N0 disease exceeds 50%, whereas it is less than 30% for those with lymph node metastases [8]. The established correlation between cancer and chronic inflammation is well-documented. Inflammatory components (e.g., cytokines, chemokines, prostaglandins, reactive oxygen species, and various transcription factors) have a proven role in facilitating tumor growth, resistance to hormonal and chemotherapy, and metastasis development [11,12,13]. This phenomenon is particularly evident in patients who are genetically predisposed and have tumor-suppressor genes and/or oncogenes. Indeed, an increase in inflammatory infiltrate at the level of the oral mucosa has been demonstrated to be associated with the malignant transformation of many potentially malignant oral disorders, including hyperkeratosis, oral lichen planus, and oral discoid lupus [12,13,14,15,16,17]. In recent years, a considerable volume of research has been conducted on using systemic hematological markers as prognostic factors in malignancies. It is frequently the case that the total number of neutrophils, lymphocytes, and platelets, either individually or when expressed as a ratio, has been linked to the prognosis of malignancies [18]. Some studies have demonstrated a higher neutrophilia rate in patients with head and neck squamous cell carcinoma (HNSCC) [19,20]. It is noteworthy that recent studies have investigated the prognostic value of the neutrophil-to-lymphocyte ratio (NLR) and platelet-to-lymphocyte ratio (PLR) in a range of malignancies. These studies have demonstrated that elevated pre-treatment ratios are associated with poorer outcomes in terms of mortality and recurrence [21,22,23,24,25,26,27].

The NLR is influenced by various factors, including age, medication use, chronic diseases (such as coronary heart disease, stroke, diabetes, obesity, psychiatric disorders, cancer of solid organs, and anemia), and stress [28,29,30,31].

It is evident that platelets are abundant in pro-inflammatory agents and are capable of releasing highly active microparticles [32]. This has been proven to play a crucial role in the development and persistence of various inflammatory diseases. In recent years, the PLR has become a prominent laboratory indicator for the prediction of neoplastic, prothrombotic, and metabolic diseases [33,34,35,36]. It has been demonstrated that variations in the PLR demonstrate a positive correlation with other systemic inflammation markers, particularly the NLR. Compared with platelet or lymphocyte counts alone, the PLR is a more reliable predictor of clinical outcomes in patients with systemic inflammation. The degree of stress-induced hypercortisolemia, which triggers platelet release into circulation and transient lymphopenia, influences the elevation of the PLR in numerous proinflammatory and prothrombotic conditions [32]. Nevertheless, this non-specific increase in the PLR can be counterbalanced by enhanced platelet destruction or consumption at sites of immune inflammation and thrombosis. Consequently, cross-checks with complete blood cell counts and additional inflammatory and immune markers are required [37].

The primary objective of this study is to assess the probability of occult cervical metastases in OTSCC based on the NLR and PLR values. This will guide the type and extent of the surgical intervention. The study’s findings should then be used to develop more efficient surgical management of the disease and to identify patients at risk of cervical metastasis.

## 2. Materials and Methods

This retrospective study involved patients treated at the Maxillofacial Surgery Unit of the Magna Graecia University of Catanzaro, Italy, between January 2002 and December 2023.

The sample was analyzed using data from the medical records archive and the archive of instrumental tests. Following the AJCC-TNM eighth edition [4] and World Health Organization (WHO) [5] guidelines, all patients were reviewed and retrospectively restaged if necessary. Glossectomies were reclassified according to Ansarin et al. [6].

**Figure 1:**
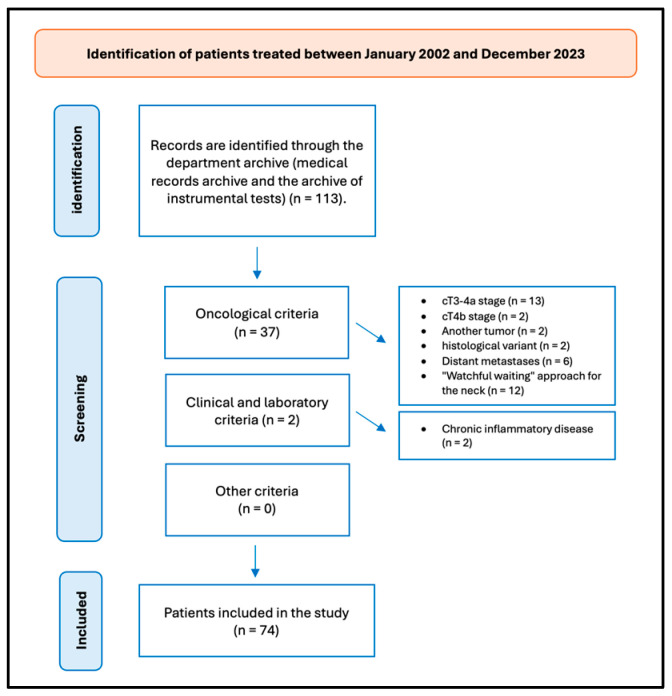
Flow chart of patient selection.

A retrospective review of patients with OTSCC was undertaken. The data were obtained from the patients’ medical records. Figure 1 presents the flow chart delineating the patient selection process.

A comprehensive anamnestic examination was conducted to ascertain any prior familiarity with neoplastic diseases, exposure to risk factors such as alcohol and smoking, and the presence of comorbidities. Additionally, a detailed general and locoregional objective examination was performed.

Subsequently, blood tests were conducted, followed by instrumental investigations, including neck and salivary ultrasound, computed tomography (CT) or magnetic resonance imaging (MRI), and positron emission tomography (PET).

### 2.1. Inclusion and Exclusion Criteria

Patients were included in the study based on the following criteria:

- Oncological criteria: patients with a histological diagnosis of primary OTSCC; patients with a negative HPV test; patients with a diagnosis of cT1-2 disease; patients with a diagnosis of cN0 disease; patients who have undergone surgery on T and N; patients who have undergone adjuvant chemotherapy and/or radiotherapy and have completed the cycles intending to achieve a curative outcome; no recurrence/persistence of disease; no other malignant tumors in different sites for at least five years (except for in situ carcinomas); patients who had undergone hospitalization and primary surgical treatment to achieve a cure; and patients with a five-year follow-up period.
- Clinical and laboratory criteria: patients not suffering from acute and/or chronic conditions, acute and/or chronic inflammatory diseases, and autoimmune hematological disorders, and under anti-inflammatory and/or steroid therapy.
- Additional criteria: patients aged 18 years or above who have provided informed consent; patients with comprehensive medical and laboratory records.

The exclusion criteria were as follows:

- Oncological criteria: patients with primary histological diagnosis other than OTSCC; patients with positive HPV test; patients diagnosed with disease cT3-4; patients diagnosed with disease cN1-2-3; patients who have undergone surgery only on T; patients undergoing neo-adjuvant chemotherapy and/or radiotherapy; presence of relapse/persistence of the disease; presence of other malignant tumors in sites other than at least five years old or ongoing; and patients with a follow-up period of less than five years.
- Clinical and laboratory criteria: patients presenting with acute and/or chronic infections, acute and/or chronic inflammatory diseases, and autoimmune hematological disorders, and under anti-inflammatory and/or steroid therapy.
- Additional criteria: patients under 18 years old, patients who have not provided informed consent, and patients with incomplete medical and laboratory records.

In the timeframe analyzed, we treated 113 patients for OTSCC, and 74 (65.49%) fulfilled the study’s inclusion criteria. Thirty-nine patients were excluded from the study for not meeting the inclusion criteria: thirteen patients were excluded from the study on account of their cT3-4a stage; two patients were excluded from the study on account of their advanced cT4b stage of OTSCC, which did not meet the criteria for surgical intervention; two patients were affected by another tumor; two patients exhibited a distinct histological variant not aligned with the one under investigation; six patients presented with distant metastases; twelve patients were excluded from the study as they had undergone surgical treatment for their primary tumors, while a “watchful waiting” approach was employed for the neck; and two patients were diagnosed with chronic inflammatory disease, which could potentially impact the blood values and reports included in the study.

### 2.2. Neutrophil-to-Lymphocyte Ratio

A normal NLR range is typically between 1 and 2, while values exceeding 3.0 or falling below 0.7 in adults are considered pathological [28]. An NLR within the grey zone of 2.3 to 3.0 may serve as an early indicator of potential pathological conditions, including cancer, atherosclerosis, infection, inflammation, psychiatric disorders, and stress. NLR is widely recognized as a reliable and cost-effective marker of cancer-related inflammation and a valuable prognostic indicator for solid tumors. Numerous meta-analyses have assessed its prognostic significance across various solid tumors, identifying an NLR cut-off value above 3.0 (IQR 2.5–5.0) [28,29].

The NLR was calculated by dividing the absolute neutrophil count by the absolute lymphocyte count.

### 2.3. Platelet-to-Lymphocyte Ratio

Recent studies on the hematological prognostic assessment of patients with OSCC reflect ongoing advancements in understanding blood-related factors influencing disease prognosis [38]. According to the findings of Zubair et al., the PLR has been demonstrated to be associated with tumor staging, lymph node infiltration, and perineural invasion. Furthermore, it has been determined that the PLR functions as a significant predictive factor for prognosis (*p* < 0.001) [39]. A PLR greater than 167.3 has been identified as an independent prognostic factor for overall survival, with a hazard ratio of 1.37 (95% confidence interval: 1.029–1.824; *p* = 0.031) [40]. As an inflammatory biomarker, mounting evidence indicates that the PLR functions as a prognostic indicator for various malignancies [41]. Elevated PLR levels may serve as a reflection of tumor-associated inflammation and immunosuppression, signifying an augmented risk of recurrence and diminished survival outcomes for patients [42].

The PLR was calculated by dividing the absolute platelet count by the absolute lymphocyte count.

### 2.4. Statistical Analysis

The pre-treatment blood data for our survey were subjected to a reprocessing procedure to calculate the NLR and the PLR. The NLR is defined as the ratio between the absolute neutrophil count and the absolute lymphocyte count, while the PLR is defined as the ratio between the platelet count and the total lymphocyte count.

To estimate the probability π(x) of metastases in cervical lymph nodes, a logistic regression model was employed, utilizing data from pre-surgery blood tests that had been previously analyzed in terms of NLR and PLR levels. The model permits the examination of the relationship between a binary dependent variable (the presence or absence of metastasis) and one or more independent variables (the neutrophil-to-lymphocyte and platelet-to-lymphocyte ratios).

The analysis was conducted using the iterative Newton–Raphson algorithm to estimate the parameters of the logistic regression model.

### 2.5. Formatting of Mathematical Components

This method is frequently employed for the estimation of parameters in maximum likelihood models:

$$\pi \left(x\right)=\frac{exp(Intercept+\beta \cdot x)}{1+exp(Intercept+\beta \cdot x)}$$

The function π(x) indicates the probability of finding either an NLR or a PLR in a cN0 category, given a specific value of NLR or PLR(x).

The probability of cervical metastasis π(x) for a given value of NLR case was calculated using the following logistic regression formula:

$$\pi \left(x\right)=\frac{1}{{1+e}^{-(\beta 0+\beta 1x)}}$$

The predicted probability of the event, designated as π(x), is the probability of lymph node metastases. The independent variable, designated as x, is the level of NLR and, subsequently, of PLR. The intercept of the logistic regression model is designated as β0, while the coefficient associated with the independent variable x is designated as β1.

The β0 intercept represents the logarithm of the probabilities (log-odds) to have lymph node metastases when the level of NLR or PLR is zero. In practice, it is the base value of the likelihood of metastasis when the level of NLR or PLR has no effect.

The β1-level coefficient shows how the lymph node metastasis probability changes for each unit increase in NLR or PLR. The likelihood of finding a lymph node metastasis in a clinically negative neck can be calculated using the following logistic regression formula:

$$\pi ((x)=\frac{exp(-1.058+0.135\cdot x)}{1+exp(-0.158+0.135\cdot x)}$$

The model’s capacity to differentiate between patients with and without cervical metastasis was evaluated using a Receiver Operating Characteristic (ROC) curve. The area under the curve (AUC) is a model performance index. A value of 1 indicates a perfect model, whereas a value of 0.5 indicates a model that does not discriminate better than the case.

## 3. Results

The clinical sample is composed of 41 males (58%) and 30 females (42%). The mean age is 63 years (range 21–95 years). The distribution by age is as follows: 4.35% in the 18–40-year range, 37.68% in the 40–60-year range, and 57.97% in the 60–95-year range. It was observed that 11 patients (15.49%) had a family history of head and neck malignancies, 22 patients (30.99%) lacked familiarity with these neoplasms, and 38 patients (53.52%) were not aware of this familiarity.

The sample under examination demonstrates the following lifestyles concerning alcohol and cigarette consumption about sex. Forty patients (56.33%) were categorized as ex-smokers, of whom sixteen had stopped smoking at the time of diagnosis of OTSCC. The remaining twenty-four had quit smoking with an average of 5.3 years before diagnosis. Nineteen (26.76%) patients had never smoked. Instead, twelve patients (16.90%) reported continuing to smoke despite having been diagnosed with OTSCC. Concerning the latter group of patients, eleven (91.67%) were male, and one (8.33%) was female. The mean number of cigarettes smoked per day was one pack, equivalent to approximately 20 cigarettes. A total of nineteen patients (26.76%) were identified as former regular consumers of alcohol. Of these, sixteen (84.21%) reported a daily consumption of one to two glasses of wine during mealtimes, while three (15.79%) reported a consumption of five to eight beers per day. Six subjects (8.45%), all of whom were male, exhibited a pattern of regular alcohol consumption even after the diagnosis of OTSCC. The mean daily alcohol consumption of the subjects was 5 beers and 2 glasses of wine at mealtimes. In addition, some of the subjects reported consuming spirits, although they were unable to specify the number of units consumed. Additionally, four subjects (5%), all men, were both smokers and regular drinkers. The mean age of smokers is 56 years, while the mean age of regular alcohol consumers is 54 years.

From a histopathological perspective, 25 patients (35.3%) exhibited evidence of lymph node metastases (pN+ group), while 46 patients (64.7%) demonstrated the absence of neck metastases (pN0 group). Table 1 shows the clinical and pathological parameters of the sample.

### 3.1. How NLR Levels Affect the Chance of Developing Metastases in the Cervical Lymph Nodes

Following the normalization of the data and the removal of any missing observations, a logistic regression model was constructed. The data from the sample under consideration are presented in Table 2. The intercept (β0) is −1.058, and the coefficient of the level of the NLR (β1) is 0.135.

**Figure 2:**
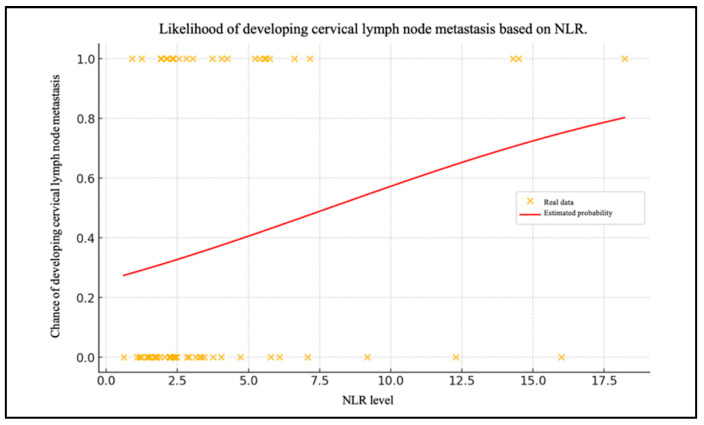
Likelihood of developing cervical lymph node metastasis based on NLR.

Chart 1 shows the patient data with their NLR levels and whether they have metastasized (0 = no, 1 = yes) (Figure 2). The red line shows the probability of lymph node metastases as a function of the NLR level, calculated using the logistic regression model.

**Figure 3:**
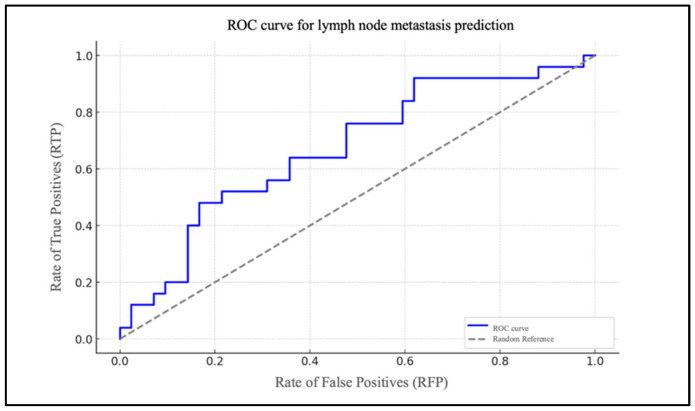
ROC curve for lymph node metastasis prediction based on NLR.

Chart 2 illustrates the ROC curve of the model, highlighted in blue (Figure 3).

It depicts the rate of true positives (RTP) in relation to the rate of false positives (RFP) for varying thresholds. The dotted grey line represents the baseline for a random classification, with an area under the curve (AUC) of 0.50. The AUC was 0.83.

### 3.2. How PLR Levels Affect the Chance of Developing Metastases in the Cervical Lymph Nodes

Following the normalization of the data and the removal of any missing observations, a logistic regression model was constructed. The data from the sample under consideration are presented in Table 3. The intercept (β0) is −1.248, and the coefficient of the level of the PLR (β1) is −0.005.

**Figure 4:**
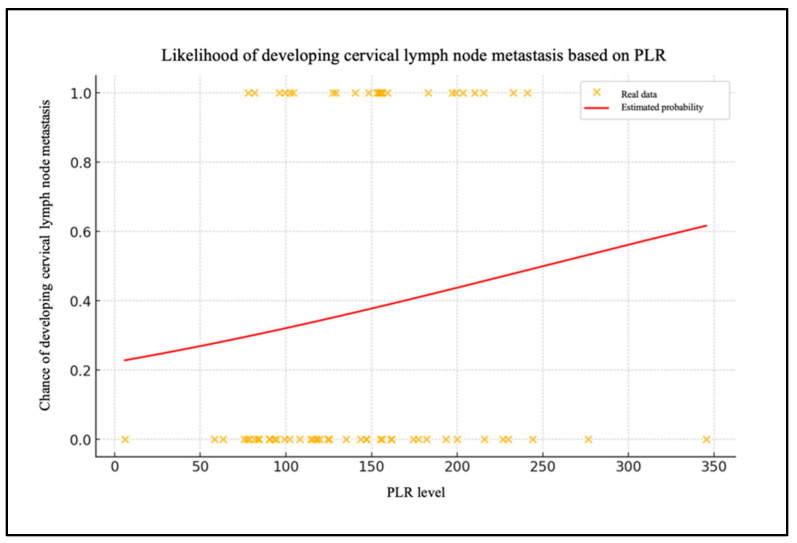
Likelihood of developing cervical lymph node metastasis based on PLR.

Chart 3 shows the patient data with their PLR levels and whether they have metastasized (0 = no, 1 = yes) (Figure 4). The red line shows the probability of lymph node metastases as a function of the PLR level, calculated using the logistic regression model.

**Figure 5:**
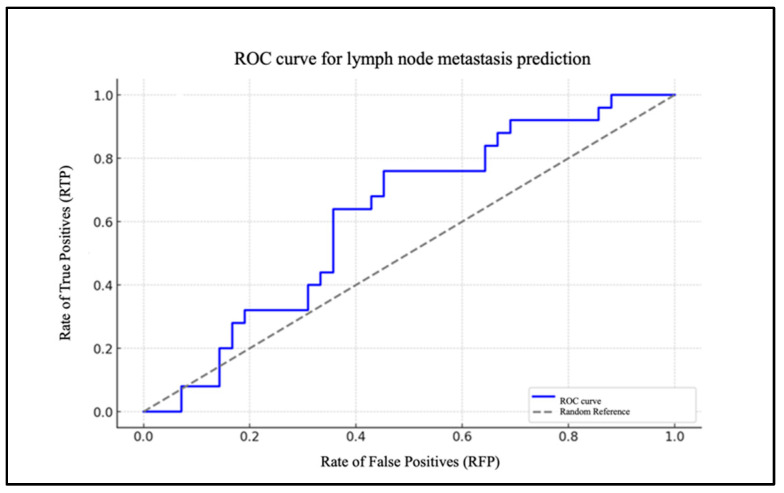
ROC curve for lymph node metastasis prediction based on PLR.

Chart 4 illustrates the ROC curve of the model, highlighted in blue, based on the PLR (Figure 5). The AUC was 0.68.

## 4. Discussion

OTSCC is the most prevalent malignancy that develops in the oral cavity [27]. Given the high propensity for local invasion and regional lymph node metastases (the incidence of this phenomenon is described in the literature to range from 8 to 46%), elective neck dissection is frequently undertaken in its early stages (T1–T2) [7,43,44] and in cN0 patients to account for the possibility of OM [43,44,45,46]. However, it is imperative that we ascertain the presence of cervical lymph node metastases to modify the treatment plan as necessary. The advancement of imaging technology has rendered CT, MRI, and PET-TC indispensable tools in the pre-operative and post-operative management of patients with OTSCC. Nevertheless, in approximately 25% of cases, OM (or micro-metastases) are already present [7,8,47], which demonstrates the limitations of imaging alone in the treatment planning of cancer patients.

Many studies have demonstrated the potential of the NLR and PLR as prognostic markers for overall survival and disease-free survival (DFS) [48,49]. The inflammatory microenvironment is a significant factor in the growth and metastasis of tumors [50]. Lymphocytopenia in cancer patients may be indicative of diffuse immunological depression [51]. A weakened immune system can have a detrimental effect on survival. The NLR and PLR may reflect the relationship between the inflammatory activating factor and the regulatory factor. Both the increase in neutrophil numbers and the decrease in lymphocyte numbers lead to the rise in the NLR. Neutrophils are frequently distributed in the tissues surrounding tumors, where they secrete elevated levels of vascular endothelial growth factor, which provides an appropriate microenvironment for promoting local tumor invasion and metastasis [52]. In contrast, lymphocytes inhibit the proliferation of cancerous cells by prompting an immune response. Tumor-infiltrated lymphocytes (TILs) consist of both T and B cells in varying proportions; TILs can often be found in the tumor stroma and within the tumor itself. The functions of these cells may be subject to change during the progression of the tumor and in response to cancer therapy. Their abundance varies according to the type and stage of the tumor and, in some cases, is related to the prognosis of the disease. The presence of TILs within tumors is frequently associated with enhanced clinical outcomes. Nevertheless, as demonstrated by numerous studies, the pro-inflammatory state can mediate a variety of processes that disrupt immune responses. Specifically, neutrophils can inhibit or even suppress the lymphocyte-mediated immune response [53]. Furthermore, this inflammatory state can stimulate platelet aggregation that protects cancer cells from the attack of TIL [54,55]. As shown by Rassouli et al. [18], the evaluation of systemic inflammatory markers (the NLR and PLR) in the preoperative period can be used as predictors of mortality and recurrence in HNSCC, irrespective of the TNM stage of development. To be more precise, the NLR is an independent predictor of recurrence, while the PLR is an independent predictor of mortality. The findings of our study corroborate those of previous research, as evidenced by the statistical analysis, which indicates that the NLR is a significant predictor of cervical lymph node metastases in patients with OTSCC.

The negative intercept value (β0= −1.058) indicates that, in the absence of the NLR level influence (i.e., with NLR = 0), the probability of metastasis is less than 50%. Consequently, without the NLR influence, the initial chance of cervical lymph node metastasis is relatively low. As the NLR value increases, the probability of metastasis also increases. This is corroborated by the positive value of the coefficient for the level of the NLR (β1 = 0.135). The probability of lymph node metastases was calculated using the NLR values.

An elevated NLR is associated with an increased likelihood of cervical metastasis, suggesting that this inflammatory marker may be useful in identifying high-risk patients.

To assess the model’s efficacy, an ROC curve was generated, demonstrating that the model exhibits a robust capacity to differentiate between patients with and without cervical metastases (AUC = 0.83). In the case of the PLR, the data are of lesser significance. The extremely low and very-close-to-zero value of the coefficient for the PLR level (β1 = 0.005) indicates that changes in the PLR levels have a minimal impact on the likelihood of metastasis. The ROC curve for the PLR report demonstrates an AUC that is situated in proximity to the random reference (hatched area in the graph), which serves to highlight the statistically insignificant predictive effectiveness of the model.

Biochemical markers are an important tool for cancer surgeons. Thus, the NLR is a very important guide to the type of treatment that should be used for the neck lymph nodes. However, our data do not show the same results for the PLR. Patients with a high NLR should be treated with neck dissection, as they are at a higher risk of developing lymph node metastases (even occult metastases).

The results of this study must be interpreted with caution due to several limitations. A significant proportion of patients are elderly or from a low socioeconomic background, which has a considerable impact on the timely diagnosis of the disease. The presence of time-worn lesions is associated with long-term local inflammation, which has the potential to interfere with the patient’s inflammatory levels. Further research is required to consider additional three-parameter inflammatory indices, such as SII and SIRI, to enhance the prediction of survival outcomes.

## 5. Conclusions

OTSCC is a common disease with an increased incidence that can often result in the production of micrometastases that are not identified by conventional instrumental examinations. Our study results demonstrate that the integration of the NLR and PLR can be utilized as a complementary framework to the TNM staging system, thereby facilitating the stratification of metastasis risk. The measurement of both the NLR and PLR can be readily incorporated into routine pre-operative evaluations, a process that is both economical and reproducible, with the added advantage of repeatability. Although the estimation of the probability of cervical metastases based on the PLR marker has yielded less significant results than that conducted on the NLR marker, the method used can probably be applied to other biomarkers or combined with additional data to enhance the model’s predictive power and facilitate the investigation of larger case studies, thereby confirming the aforementioned results.

## Figures and Tables

**Table 1:** The clinicopathological parameters of the sample.

		pN0 Group (46 Cases)	pN+ Group (25 Cases)
Gender	Male Female	30 16	11 14
Age	Range Mean	32–95 72 y.o.	21–67 45 y.o.
Smokers	Yes No	7 39	9 16
Alcohol	Yes No	4 42	6 19

**Table 2:** NLR values in eligible patients.

Metastases in the Cervical Lymph Nodes	NLR Average	NLR Minimum Value	NLR Maximum Value
pN0	3.69	0.62	16
pN+	3.84	0.91	18.21

**Table 3:** PLR values in eligible patients.

Metastases in the Cervical Lymph Nodes	PLR Average	PLR Minimum Value	PLR Maximum Value
pN0	175.22	96.53	215.01
pN+	162.98	92.93	208.38

## Data Availability

The data are available upon request from the corresponding author, for privacy reasons.
